# Immunogenetic diversity of MHC class II B-Lβ genes in Brazilian Caipiras (free-range) chickens laying blue eggs

**DOI:** 10.3389/fimmu.2025.1644110

**Published:** 2025-08-21

**Authors:** Carlos André da Veiga Lima Rosa Costamilan, Ediane Paludo, Marcos Edgar Herkenhoff, Fábio Pértille, André Luigi Soares de Souza, Carolina Rosai Mendes, Guilherme Dilarri

**Affiliations:** ^1^ Department of Fisheries Engineering and Biological Sciences, Santa Catarina State University (UDESC), Laguna, Santa Catarina, Brazil; ^2^ Institute of Improvement and Molecular Genetics (IMEGEM), Santa Catarina State University (UDESC), Lages, Santa Catarina, Brazil; ^3^ Physiology and Environmental Toxicology Program, Department of Organismal Biology, Evolutionary Biology Center, Uppsala University, Uppsala, Sweden; ^4^ Multicentric Graduate Program in Biochemistry and Molecular Biology (PMBqBM), Santa Catarina State University (UDESC), Lages, Santa Catarina, Brazil

**Keywords:** B-Lβ genes, blue egg, Caipira chicken, genetic variability, major histocompatibility complex (MHC)

## Abstract

**Introduction:**

Brazilian Caipira chickens that lay blue eggs are known to possess unique genetic traits. This study investigates the immunogenetic diversity of MHC class II B-Lβ genes (B-LβI and B-LβII) in this population, aiming to assess their potential value in selective breeding programs focused on disease resistance.

**Material and methods:**

A total of 100 chickens were analyzed using targeted sequencing of the B-LβI and B-LβII genes. The resulting nucleotide sequences were evaluated for polymorphism and compared with known alleles described in previous studies.

**Results:**

Fifteen unique nucleotide sequences were identified, of which five had not been previously reported in the scientific literature. The new alleles exhibited significant polymorphism, confirming high genetic diversity within the population.

**Discussion:**

The genetic variability observed supports earlier findings regarding the diversity of MHC genes in local chicken breeds. These novel alleles may confer advantages in immune responsiveness, reinforcing the importance of preserving local breeds as reservoirs of functional genetic diversity.

**Conclusion:**

Brazilian Caipira chickens laying blue eggs display remarkable immunogenetic diversity in MHC class II B-L β genes. This variation includes novel alleles with potential application in future breeding programs. Conservation and utilization of this genetic resource can contribute to the development of healthier and more disease-resistant commercial chicken lines.

## 1 Introduction

Domestic chickens (*Gallus gallus domesticus*) are native to Southwest Asia and primarily descended from a wild bird known as the Red Jungle Fowl (*Gallus gallus*) (1). They were first introduced to Brazil by European navigators. These navigators brought breeds from Eastern, Mediterranean, and Southern Europe, which were roamed freely on farms. This freedom led to random crossbreeding among the chickens, resulting in the Brazilian chicken ecotype known as “Caipiras”, popularly called free-range chickens. Some Brazilian Caipira chickens are known for laying blue eggs. This unique eggshell coloration, which can range from blue to green, is characteristic of a South American breed called Araucana. The Araucana chickens originate from the Arauca region in Chile and are one of only two breeds that naturally lay blue eggs (2). These blue-egg-laying birds spread throughout Brazil and, through further mixing, contributed to the development of the national free-range chickens, thus giving rise to the Brazilian Caipiras chickens that lay blue eggs.

The originality and broad genetic variability of chickens make them an excellent model for exploring genetic traits, particularly those associated with immune function. Among these traits, immune-related genes on chromosome 16 stand out. These genes, part of the major histocompatibility complex (MHC), are among the most polymorphic regions in the chicken genome and serve as an ideal model for studying genetic diversity (3). The immune response in chickens to external pathogens is initiated by presenting antigens from these pathogens to the immune system’s effectors cells. This action is performed by antigen-presenting cells, including B lymphocytes, macrophages, and a small percentage of T lymphocytes (4). These cells have surface molecules called glycoproteins that bind to these antigens. These glycoproteins, known as MHC of class II, are formed by two polypeptide chains, one α and one β (5). The MHC is organized into two independent and polymorphic gene clusters, called the B *locus* and the Y *locus* (6). The α chain is encoded by the B-Lα gene, which exists in a single copy within the chicken genome, located at the B *locus*, and is monomorphic (7). In the genome of these chickens, there are five genes that can encode the β chain polypeptide. At the B *locus*, two genes for the β chain are located, B-LβI and B-LβII, and at the Y *locus*, Y-LβIII, Y-LβIV and Y-FβV; where the B-LβI and B-LβII genes are the most highly expressed, the other three (Y-LβIII, Y-LβIV and Y-LβV) have low or no expression (7). The class II molecules of chickens encode the B-Lα gene and one of the B-Lβ genes. In these chickens, the B-Lα gene is monomorphic, meaning it does not vary among individuals. The differences observed in the class II molecules, for variations in the immune response arise from the different alleles of the B-Lβ genes present in their genomes. To date, 96 alleles have been identified for the B-LβI and B-LβII genes (8).

This notable genetic variability of immune-related genes is particularly relevant when examining local breeds such as the Brazilian “Caipiras” (*Gallus gallus domesticus*), known for laying blue eggs, originated from random crosses of various chicken breeds brought to Brazil during early colonization, including Araucanian chickens. This Brazilian Caipira chicken subspecies is known for its high genetic variability, low productivity, and remarkable rusticity, which includes increased resistance to diseases (such as bacteria of genus *Salmonella*), as well as adverse climate, temperature, and feeding conditions (9–13). Since they have not undergone artificial selection, these chickens exhibit greater genetic diversity than modern commercial breeds, which have been significantly improved through genetic programs (10, 12). This high genetic variability of Caipiras chickens has already been proven for gene loci of the chickens’ immune system when analyzing the two B-F genes of Brazilian Caipiras chickens that lay blue eggs (12). A sample of 100 chickens revealed 23 alleles, ten of which have not been described in commercial chicken breeds (12). If there is this large allelic variation for the B-F genes, we can expect that a similar variation should be present for other genes related to the immune system of chickens, such as the B-Lβ genes, in Brazilian Caipiras chickens.

Therefore, this study aimed to investigate the variability of the B-LβI and B-LβII genes in Brazilian Caipira chickens that produce blue eggs. There has been no investigation into the polymorphism of these genes in this type of chicken. This study aimed to offer a deeper understanding of the immunological genetic mechanisms involving these animals. For that reason, these analyses are crucial for understanding the variation in chickens regarding loci associated with immunity to external microorganisms, enabling, in the future, the increase of this immunity in these birds.

## 2 Materials and methods

### 2.1 Brazilian Caipiras chickens

In this study, peripheral blood samples were obtained from 100 Brazilian Caipiras chickens that lay blue eggs, which were preserved at -20°C using ethylenediaminetetraacetic acid (EDTA) as an anticoagulant. The samples were collected from the rural region of Dois Lageados, RS, Brazil. DNA extraction was carried out with the GeneJET™ kit (Whole Blood Genomic DNA Purification Mini Kit) from Thermo Scientific™ (Waltham, Massachusetts, USA). The collection process began with the hatching of embryonic blue eggs, and after that, the chicks hatched were kept in captivity until the blood samples were collected.

### 2.2 DNA extraction and amplification

Genomic DNA was isolated from peripheral blood using the protocol established by Sambrook et al. (14) using the GeneJET™ kit from Thermo Scientific™ (Waltham, Massachusetts, USA). The desired fragments were amplified using the Polymerase Chain Reaction (PCR) technique, as described by Li et al. [15], from the isolated DNA. The primers used were: 5’-CGTTCTTCTTCTRCGGTRBGAT-3’ (sense; primer 1), 5’-GCTCCTYTGCACCGTGAAGG-3’ (antisense; primer 3) (14–16), and 5’-GTGCCCGCAGCGTTCTTC-3’ (sense; primer 2) (16), corresponding to a region of the beginning of exon 2 (primer 1), end of intron 2 and beginning of exon 2 (primer 2) and to a sequence of the end of exon 2 (primer 3). The result of the amplification is a 267 base pair (bp) fragment for the fragment amplified by primers 1 and 3, and a 277 base pair fragment for the fragment amplified by primers 2 and 3.

The PCR reagent mixture contains: 100 ng of DNA, 2.5 µL of 10× buffer (100 mM Tris-HCl, pH 8.3, 500 mM KCl, 15 mM MgCl2), 2.5 µL of PCR × Enhancer System, 2 µL of dNTP mixture (1.25 mM dATP, dCTP, dGTP and dTTP - phosphated deoxyribonucleotides), 0.2 µM of each primer, 1 U of TaqDNA polymerase Platinum from Invitrogen Life Technologies (Carlsbad, CA, USA); and completed with ultra-pure water to obtain a final volume of 25 µL. Amplification consisted of 2 minutes of initial denaturation at 95°C, followed by 35 cycles of denaturation at 95°C for 1 minute, annealing at 61°C for 5 seconds, extension at 72°C for 2 minutes, and final extension at 72°C for 10 minutes.

The total amplification was subjected to electrophoresis in 1% agarose gel stained with ethidium bromide and visualized under ultraviolet light. A cut was made in the gel to remove the section containing the expected DNA amplification, which was approximately 200–300 mg. The amplified DNA was then extracted from the gel using the HiYield™ Gel/PCR DNA Extraction Kit from Real Biotech Genomics (Banqiao City, Taiwan).

### 2.3 Cloning and sequencing of amplified DNA

The amplified DNA fragment removed from the gel was cloned using the TOPO-TA Cloning Kit for Sequencing from Thermo Scientific™ (Waltham, Massachusetts, USA). After inserting the DNA fragment into the plasmid and then into the bacterial cells of *Escherichia coli*, we seeded the cells containing the insert onto solid culture medium Agar BL (Lennox L Agar), which included the antibiotic Ampicillin. This selection allowed for the growth of only those cells that carried the plasmid. The cells were grown for approximately 16 hours at 35°C. Then, the desired colonies of *E*. *coli* were selected and placed in Lennox L Broth liquid medium containing Ampicillin at 20 µg mL^-1^. Next, the bacterial cells were centrifuged and purified with the PureLink™ Quick Plasmid Miniprep Kit from Thermo Scientific™ (Waltham, Massachusetts, USA).

Then the PCR reaction was performed with the purified plasmid samples. The PCR reagent mixture contained: approximately 100 ng of amplified DNA, 2.5 µL of 10X buffer (100 mM Tris-HCL, pH 8.3, 500 mM KCL, 15 mM MgCl2), 2 µL of dNTP mixture (1.25 mM dATP, dCTP, dGTP and dTTP), 0.2 µM of each primer (the same ones used in the initial amplification), 1 U of Platinum TaqDNA polymerase from Invitrogen Life Technologies (Carlsbad, CA, USA), and completed with ultra-pure water to obtain a final volume of 25 µL. The amplification procedure included an initial denaturation step of 2 minutes at 95°C, followed by 35 cycles consisting of denaturation for 1 minute at 95°C, annealing for 30 seconds at 61°C, and extension for 2 minutes at 72°C. A final extension was performed at 72°C for 10 minutes. The PCR products were purified using the enzymes Shrimp Alkaline Phosphatase (SAP) and Exonuclease I (EXO I) from Fermentas Life Sciences (Burlington, Canada). The purification process involved incubating the samples at 37°C for 30 minutes and then at 80°C for 15 minutes to remove unused primers and oligonucleotides labelled with fluorophore, undesirable for sequencing.

The sequencing was performed on the ABI Prism 3130 genetic analyzer (Applied Biosystems Life Technologies - Waltham, Massachusetts, USA) using the ABI Prism BigDye Terminator Cycle Sequencing Ready Reaction V3.1 kit (PerkinElmer Applied Biosystems - Waltham, Massachusetts, USA), labelling with fluorescent terminators. During data analysis, the obtained sequences were manually corrected using the Chromas 1.45 program and aligned with the Clustal W program (17, 18). These sequences were then compared to those already described in the NCBI database (National Center for Biotechnology Information).

### 2.4 Amino acid comparison and allelic denomination

Amino acid sequences were obtained from the Mega 5 software. The alignment used as standard BLB1*12 (B-LB12major; GenBank accession n° AJ248576) and BLB2*12 (B-LB12c; GenBank accession n° AJ248578) as described by Jacob et al. (19).

The system used to apply allelic nomenclature to the sequences was an acronym for the name of the locus (BLB) and an acronym for Caipiras chickens (CC), followed by a number corresponding to each of the B-LβI and B-LβII loci, for example BLB*CC1a or BLB*CC1b, respectively.

## 3 Results

### 3.1 Nucleotide sequences

DNA samples were collected from 100 Caipira chickens that lay blue eggs, then amplified for the region of interest and analyzed. The animals were grouped according to the exon 2 sequence. Then, 16 animals were selected, ensuring at least one representative from each group for the DNA amplification reaction, cloning, and sequencing. The samples were subjected to the primers of the B-LβI and B-LβII genes. For each nucleotide sequence (allele) of the B-Lβ genes identified, we sequenced three clones from at least two different animals, resulting in a total of six sequences for each allele. From these 16 chickens, we obtained 15 different sequences: eleven B-LβII sequences and four B-LβI sequences. Comparing, the 15 samples obtained from B-Lβ with the literature, it can be observed that ten are already described in the NCBI. The other five sequences are new and have not yet been described in the literature or included in the NCBI database. The ten sequences already in the NCBI database have the following accessions: AF099115, AB426150, AY228553, M87655, GGU76305, EU579528, AY744361, AJ248586, AJ248575, and AF539401 (**Table 1**).

**Table 1:** Sequence names associated with Brazilian Caipira chickens that lay blue eggs, along with their corresponding sequences from the literature.

Sequence names	Literature Access (NCBI)
B-LB*CC 1b	M87655
B-LB*CC 2b	AY744361
B-LB*CC 3b	AF099115
B-LB*CC 4b	GGU76305
B-LB*CC 5b	EU579528
B-LB*CC 6b	“New”
B-LB*CC 7b	AY228553
B-LB*CC 8b	AB426150
B-LB*CC 9b	“New”
B-LB*CC 10b	“New”
B-LB*CC 11b	“New”
B-LB*CC 1a	AF539401
B-LB*CC 2a	AJ248575
B-LB*CC 3a	“New”
B-LB*CC 4a	AJ248586

The acronym BLB refers to the locus (B-Lβ), CC denotes Caipira chickens, and each locus is followed by a number indicating B- LβI or B-LβII (for example, BLB*CC1a or BLB*CC1b).

Out of the eleven amplified B-LβII sequences, seven matched sequences previously reported in the literature, while four were identified as “new” sequences (**Table 1**). Among the four B-LβI sequences discovered, three had been documented before, but one was previously unreported and thus classified as a new one (**Table 1**). When comparing the “new” sequences with those already described, it was observed that the BLB*CC9b sequence aligned with the sequence of accession DQ008585 and presented nine specific differences. The B-LB*CC3a sequence aligned with the HQ218318 sequence, with seven differences. The B-LB*CC10b sequence aligned with the AB426150 sequence, with five different points. The B-BL*CC6b sequence aligned with the AF099115 sequence, with four specific differences. The B-LB*CC11b sequence aligned with the HQ203725 sequence, with two individual differences.

**Figure 1:**
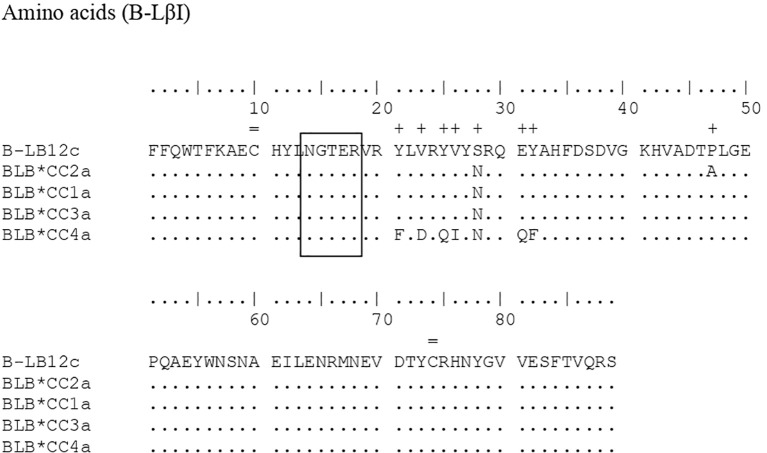
Comparison of eleven nucleotide sequences of the B-LβII gene from Brazilian Caipira chickens that lay blue eggs, against the standard sequence B-BL12major (AJ248576). A (+) mark is placed above the standard sequence, indicating the point of differentiation between the sequences observed in the Caipira chickens that lay blue eggs and the standard sequence.

**Figure 2:**
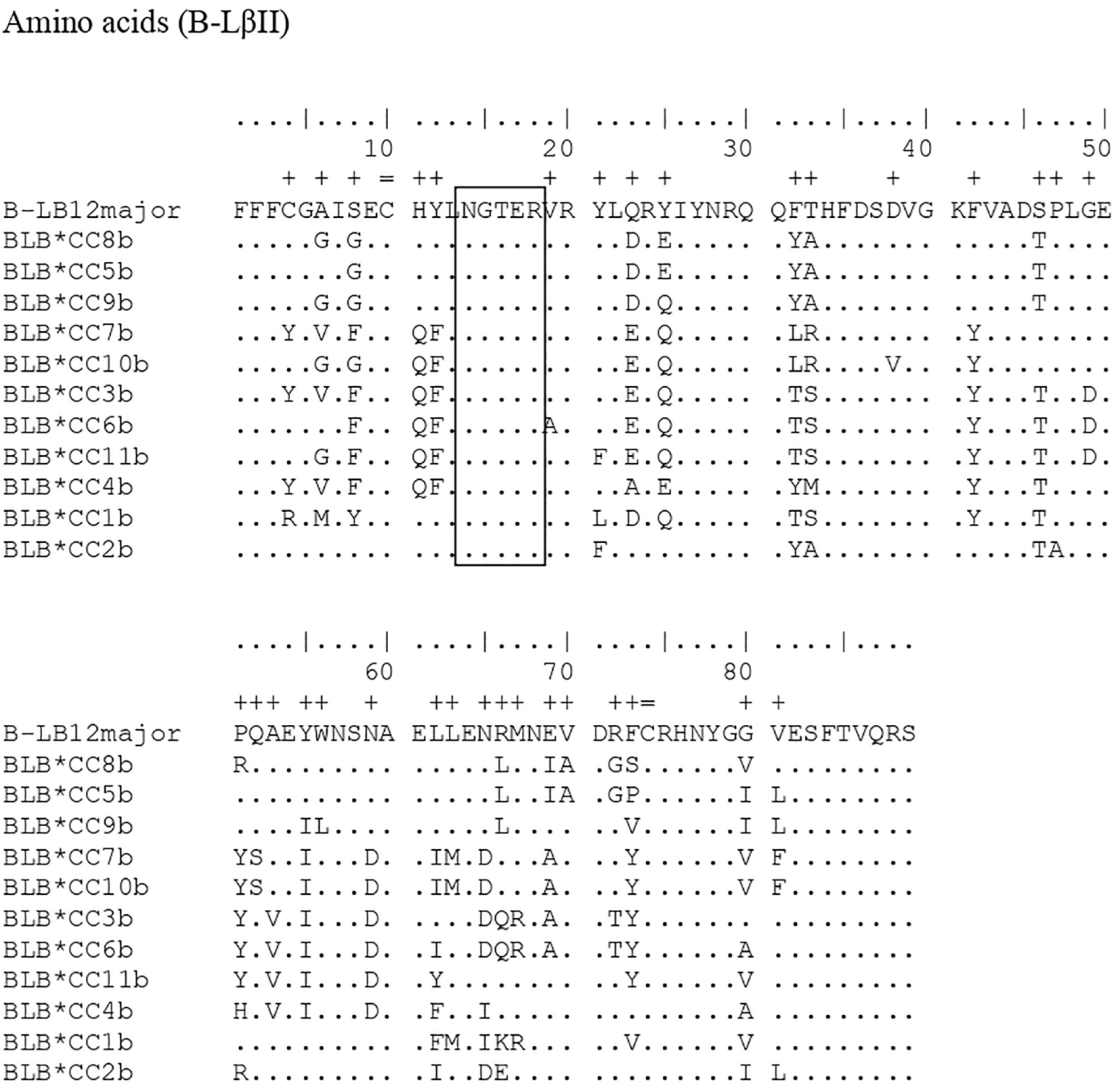
Comparison of the nucleotide sequences of the B-LβI gene from Brazilian Caipira chickens that lay blue eggs, alongside the standard sequence B-BL12c (AJ248578). A (+) mark is placed above the standard sequence, indicating the point of differentiation between the sequences observed and the standard.

**Figures 1**, **2** compare the nucleotide sequences from this study with those of two standard alleles, highlighting differences with a symbol (+) in the relevant positions. For the sequences found in the present work referring to the B-LβII gene, the standard sequence B-LB12major (AJ248576) was used, and the differences found were in the positions: 11, 16, 17, 22-24, 33, 35, 39, 56, 60, 62, 63, 67, 69, 73, 75, 93-98, 113, 125, 126, 129, 132, 136, 139, 146, 151-156, 158, 159, 163, 164, 166, 175, 184, 185, 187, 192-197, 200, 204-207, 209, 214, 215, 217, 218, 238, 239, 241, 243, and 246. For the B-LβI gene, the standard sequence was B-LB12c (AJ248578), and the differentiation positions were found in: 24, 62, 68, 73, 75, 76, 83, 91, 95, 132, and 139.

The new sequences were deposited in the GenBank accession numbers for each nucleotide sequences B-LB*CC 6b access PV637473; B-LB*CC 9b access PV637474; B-LB*CC 10b access PV637475; B-LB*CC 11b access PV637476; B-LB*CC 3a access PV664779.

### 3.2 Amino acid sequences

**Figure 3:**
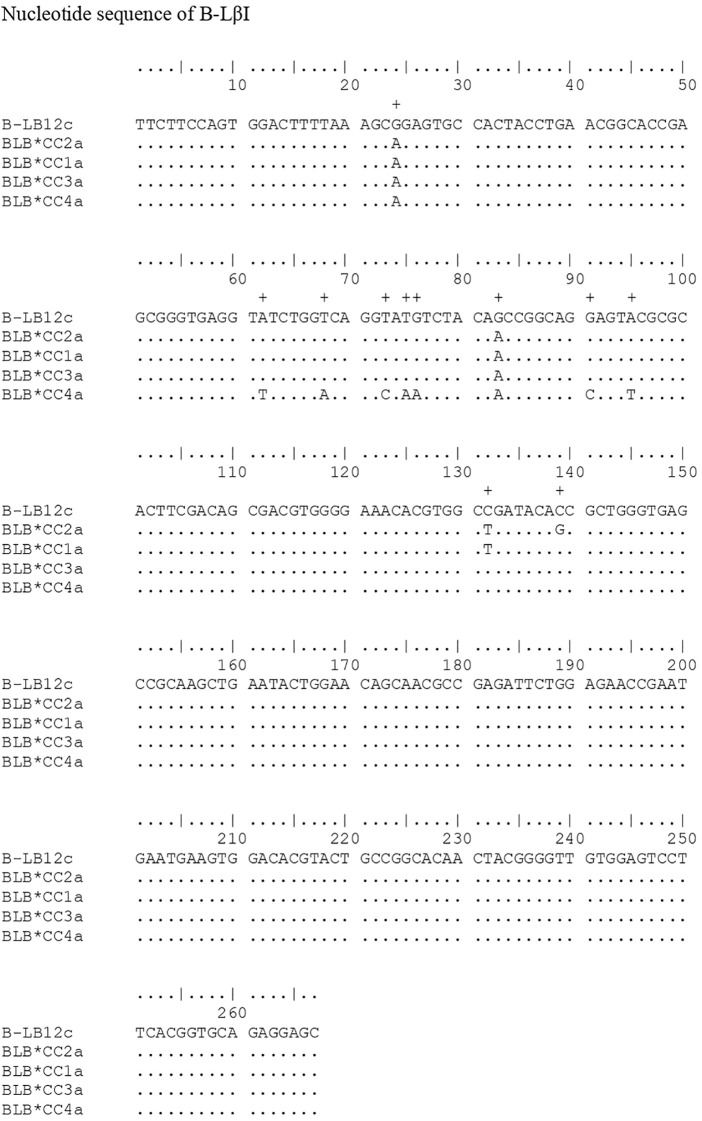
Comparison of the eleven polypeptide sequences obtained from the simulation transformation of the nucleotide sequences described in this work with the standard sequence B-BL12major (AJ248576), also transformed. A plus sign (+) is indicated above the standard sequence, highlighting the points of differentiation between the observed sequences and the standard. In the amino acid sequences, the conservation of some specific binding sites is observed: in positions 14-18 (-NGTER-) with a rectangle around the sequence, the carbohydrate binding site (CHO); in positions 10 and 74, the disulfide band (S-S) represented by the symbol =.

The 15 nucleotide sequences found were translated into amino acid sequences. **Figure 3** shows the regions with differentiation sites in their positions 4, 6, 8, 11, 12, 19, 21, 23, 25, 32, 33, 38, 42, 46, 47, 49, 51-53, 55, 56, 59, 62, 63, 65-67, 69, 70, 72, 73, 80, and 81; which are from the amino acids generated for the B-LβII gene, using the BLB12c - AJ248576 sequence as a comparison standard.

**Figure 4:**
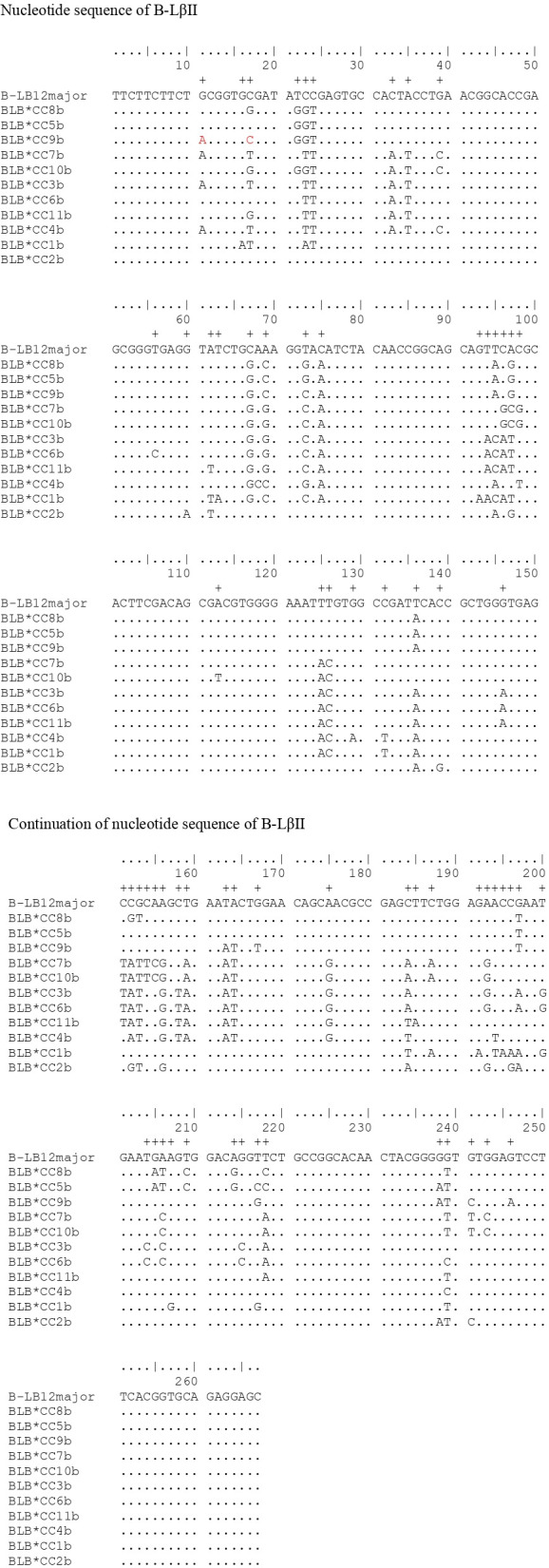
Comparison of the four polypeptide sequences obtained from the simulation transformation of the nucleotide sequences described in this work with the standard sequence B-BL12c (AJ248578). Above the standard sequence, a marking (+) is found, referring to the point of differentiation between the sequences found and the standard. In the amino acid sequences, the conservation of some specific binding sites is observed: in positions 14-18 (-NGTER-) with a rectangle around the sequence, the carbohydrate binding site (CHO); in positions 10 and 74, the disulfide band (S-S) represented by the symbol =.

In **Figure 4**, the amino acid sequences relating to the B-LβI gene can be observed. The sequence shows significantly less nucleotide variability, making it more conserved than the amino acid sequences of the B-LβII gene. When aligned with B-LB12c (AJ248578), similarities were among the sequences. However, the sequence BLB*CC4a exhibited more significant variations (AJ248586) and differed from the standard sequence at six specific positions: 21, 23, 25, 26, 28, 31, and 32. Notably, at position 28, all sequences analyzed diverged from the standard sequence. Also, in all the amino acid sequences obtained, conservation of some specific binding sites was observed: in positions 14-18 (-NGTER-), carbohydrate binding site (CHO); and in positions 10 and 74, the disulfide bridge (S-S).

## 4 Discussion

**Figure 5:**
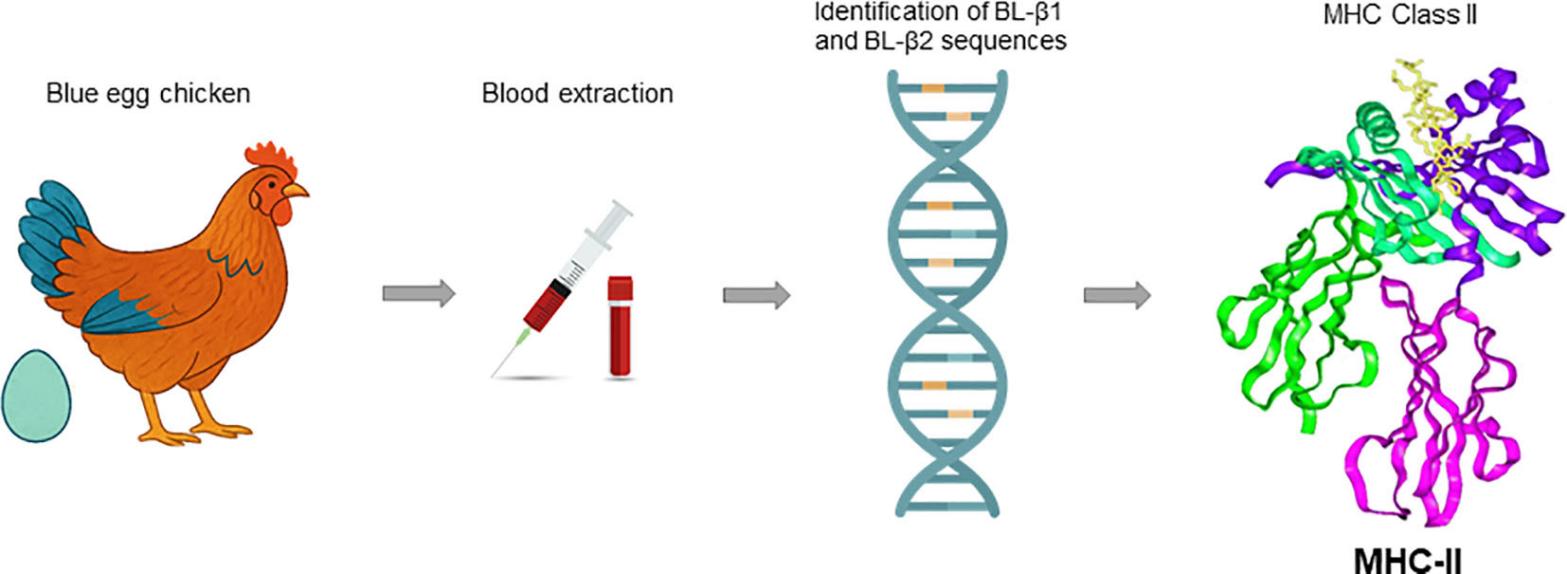
Process of genetic analysis in blue egg chickens. The diagram illustrates the stages from blood extraction to the identification of BL-β1 and BL-β2 sequences, concluding with the depiction of the MHC Class II structure.

The MHC class II genes of chickens and other animals, are characterized by high genetic variability (3). Numerous studies have demonstrated that the diversity and genotypes of the Major Histocompatibility Complex (MHC) are critical determinants of disease resistance in chickens (**Figure 5**). Brazilian Caipira chickens display even greater genetic diversity than commercial lines, primarily because they have not been subjected to artificial selection pressures (12). This broader genetic repertoire provides an immunological advantage, enhancing their ability to respond to a wide range of pathogens, including those that commonly affect commercial poultry (20–23). Supporting these findings, the present study identified 15 distinct alleles of the B-LβI and B-LβII genes in a sample of 100 individuals from a single population of blue-egg-laying Brazilian Caipira chickens. Out of the 15 identified alleles, eleven are derived from the B-LβII gene, while four are associated with the B-LβI gene, highlighting the high polymorphism in the MHC genes of chickens. This pattern suggests a unique co-evolutionary dynamic for these genes and reinforces the potential for MHC allele diversity in indigenous or free-range chicken populations. In contrast, commercial chickens exhibit limited MHC allelic diversity, resulting in intensive artificial selection (20–22), has been associated with reduced immune competence in these birds (21). This statement supports the findings of Martin et al. (22), who conducted a comprehensive study on the global population genetics of the MHC in chickens; where our work has confirmed the identification of five additional new MHC sequences in Brazilian free-range chickens, demonstrating the allelic variability of the MHC gene in these birds, confirming the affirmations of Martin et al. (22) about the diversity in MHC class I and II alleles.

A similar pattern of MHC polymorphism was reported by Lima-Rosa et al. (24). They studied Brazilian Caipiras chickens that lay blue eggs, specifically focusing on the B-FI and B-FIV genes, which are part of the MHC class I. They identified 23 sequences of the B-F gene from 100 chickens, including 13 new sequences that had not been described yet (24). Recent studies on non-commercial birds have shown similar polymorphism (25, 26). Vatankhah et al. (26) revealed a significant impact of the MHC allele due to this polymorphism in broiler chickens. Yuan et al. (3) also studied the genetic diversity of the MHC B-F/B-L in various chicken breeds, including Chinese domestic chickens, Red Jungle Fowl, Cornish, and White Leghorn chickens. Their analysis confirmed a high level of genetic diversity, particularly among Chinese domestic breeds, suggesting that these breeds possess a broad-spectrum resistance to pathogens. Intensive long-term selection of commercial chicken breeds has significantly decreased the diversity of the B-F/B-L region of the MHC (3). This finding supports the observation that commercial chicken breeds have lower polymorphism than non-commercial birds (27), particularly in the haplotypes of the B-LβII gene, as in this study. Worley et al. (28) successfully identified specific sequences of MHC genes in both classes (I: B-FI and B-FII; II: B-LβI and B-LβII) in red-necked chickens. Out of 84 samples analyzed, nine sequences of the B-F gene were found (three of B-FI and six of B-FII) and ten sequences of the B-Lβ gene (four of B-LβII, five of B-LβI, and one undefined) (28). This high genetic variability for the B-LβII gene is also confirmed in Brazilian Caipiras chickens that lay blue eggs, as proved in the present work.

Many studies have examined the B-Lβ genes in chickens 96 different alleles have been identified: 14 for the B-LβI gene and 82 for the B-LβII gene. In the current study, seven sequences previously documented in the literature were identified, representing 8% of the known alleles for the B-LβII gene, along with four new sequences. For the B-LβI gene, the study uncovered three previously described sequences, which account for 21% of the total sequences available in the literature, plus one new sequence. Therefore, although the number of B-LβI gene sequences found in this study is relatively small, it is proportionally greater than the number of sequences for the B-LβII gene. The fewer alleles recorded for the B-LβI gene in the also indicate a lower level of genetic variability for this gene than B-LβII.

In the B-LβII sequences, alignment with the standard sequence B-LB12major (AJ248576, **Figure 1**) reveals several variant positions. Ten positions with two variants each: 132, 154, 194, 196, 204, 205, 209, 214, 215, and 243. Nine positions with three variants: 11, 39, 62, 98, 146, 187, 200, 217, and 238. Finally, three positions exhibit four variants: 22, 94, and 158. In a similar study, Xu et al. (29) identified 32 positions with two variants, 17 positions with three variants, and two positions with four variants in MHC B-LβII in Chinese indigenous chickens. In the current study, after aligning B-LβI amplification with the standard sequence B-LB12c (AJ248578), we found two positions with four variations — positions 24 and 83. One position with two variations at position 132, and eight positions with a single variation each at positions 62, 68, 73, 75, 76, 83, 91, 95, and 139. In the alignment of the polypeptides generated for the B-LβII gene, there are 33 positions where the sequences differ from the standard sequence B-BLmajor. These positions of differentiation are as follows: 4, 6, 8, 11, 12, 19, 21, 23, 25, 32, 33, 38, 42, 46, 47, 49, 51-53, 55, 56, 62, 63, 65-67, 69, 70, 72, 73, 80, and 81. For B-LβI, eight differentiation points were identified: positions 21, 23, 25, 26, 28, 31, and 32, compared to the standard tape B-LB12c (AJ248578). At position 132, there is a difference in the nucleotide sequence between samples B-LB*CC2a and B-LB*CC1a. However, this difference does not affect the resulting polypeptides at position 44, since the nucleotide C (cytosine) that is in the standard sequence and the T (thymine) that is in the sequences found in the work encode the amino acid alanine. Therefore, there is no change in the amino acid sequences. Similar to other studies involving MHC genes in chickens (8, 28, 29), a specific set of amino acids was to remain constant in positions 14-18 (-NGTER-). This sequence corresponds to the carbohydrate-binding site, making it crucial for the molecule’s function. As a result, no variations were observed in this area, which is consistent with the findings of the present study. The same applies to another crucial point of the molecule, the disulfide bridges at positions 10 and 74, which are also conserved, as proved in this work.

Identifying the 15 alleles in a sample of 100 Brazilian Caipira chickens that lay blue eggs highlights a remarkable degree of genetic variability. These findings are consistent with the genetic diversity previously reported by Lima-Rosa et al. (12, 24). The alleles identified in this study represent a valuable genetic resource that could be leveraged in selective breeding programs, improving disease resistance in commercial chicken lines.

## 5 Conclusion

Brazilian Caipiras (Free-range) chickens that lay blue eggs showed significant polymorphism for MHC class II molecules (B-LβI and B-LβII). Through the sequencing technique of the B-LβI and B-LβII genes, it was possible to detect 15 different nucleotide sequences in a sample of 100 animals. Among the 15 sequences analyzed, 5 had not been previously described in the literature. These findings confirm the significant genetic variability in the Brazilian Caipiras chickens that lay blue eggs regarding the B-FI and B-FIV genes (MHC class I). The main importance of obtaining these “new” alleles lies in their potential future applications, particularly in genetic improvement programs, enhancing disease resistance. These programs must preserve polymorphism at loci where variation is essential, especially for traits related to disease resistance. They will indirectly enhance both production and the birds’ ability to withstand pathogens.

## Data Availability

The datasets presented in this study can be found in online repositories. The names of the repository/repositories and accession number(s) can be found in the article/supplementary material.
